# Supplementary material for: Specific pharmacological and Gi/o protein responses of some native GPCRs in neurons
Source: Nat Commun. 2024 Mar 5;15:1990. doi: 10.1038/s41467-024-46177-z (PMC10914727; doi:10.1038/s41467-024-46177-z)
Supplement: Supplementary file 1 — Supplementary Information [file 41467_2024_46177_MOESM1_ESM.pdf]

Specific pharmacological and $G_{i/o}$ protein responses of some native GPCRs in neurons

Chanjuan Xu[a,b,#], Yiwei Zhou[a,#,d], Yuxuan Liu[a,#], Li Lin[a], Peng Liu[a], Xiaomei Wang[a], Zhengyuan Xu[a], Jean-Philippe Pin[c,*], Philippe Rondard[c,*], Jianfeng Liu[a,b,*]

[a] Cellular Signaling Laboratory, Key Laboratory of Molecular Biophysics of Ministry of Education, College of Life Science and Technology, Huazhong University of Science and Technology, Wuhan, Hubei, China

[b] Bioland Laboratory, Guangzhou Regenerative Medicine and Health Guangdong Laboratory, 510005 Guangzhou, China

[c] Institut de Génomique Fonctionnelle (IGF), Université de Montpellier, CNRS, INSERM, 34094 Montpellier, France

[#] These authors contributed equally to this work.

[d] Present address: Kindstar Global Precision Medicine Institute, Wuhan, China

* To whom correspondence may be addressed. Email: jean-philippe.pin@igf.cnrs.fr, philippe.rondard@igf.cnrs.fr or jfliu@mail.hust.edu.cn

Supplementary Tables

Supplementary Table 1. Potency of the indicated ligands of the $GABA_B$ receptor in the different neurons and in HEK293 cells using the indicated $G_{i/o}$ protein sensors. Values are mean ± SEM from n independent experiments as indicated.

		pEC$_{50}$ / pIC$_{50}$ ± SEM (n)						
		CGP64213	GABA	baclofen	SKF 97541	APPA	baclofen + Rac BHFF 1 μM	baclofen + Rac BHFF 10 μM
CGNs	G_{i1}	7.55 ± 0.04 (5)	5.03 ± 0.07 (5)	5.32 ± 0.06 (11)	5.63 ± 0.04 (5)	6.13 ± 0.14 (3)	5.56 ± 0.03 (3)	6.19 ± 0.10 (3)
	G_{oA}	7.59 ± 0.11 (6)	5.07 ± 0.02 (3)	5.57 ± 0.06 (4)	5.69 ± 0.12 (3)	6.16 ± 0.14 (4)	5.15 ± 0.10 (3)	5.85 ± 0.09 (3)
Cortical neurons	G_{i1}		5.58 ± 0.03 (4)	5.95 ± 0.03 (5)	6.52 ± 0.10 (3)			
	G_{oA}		5.38 ± 0.09 (3)	5.75 ± 0.08 (4)	6.22 ± 0.13 (3)			
Hippocampal neurons	G_{i1}		5.48 ± 0.05 (3)	5.99 ± 0.04 (4)	6.64 ± 0.04 (3)			
	G_{oA}		5.15 ± 0.14 (3)	5.58 ± 0.12 (3)	6.16 ± 0.06 (3)			
HEK293 cells transfected $GABA_BR$	G_{i1}		6.47 ± 0.01 (3)	6.08 ± 0.10 (3)	7.14 ± 0.09 (3)	7.67 ± 0.09 (3)		
	G_{oA}		6.45 ± 0.04 (3)	6.07 ± 0.07 (3)	7.07 ± 0.08 (3)	7.77 ± 0.10 (3)		

Supplementary Table 2. Potency and efficacy of the indicated orthosteric ligands on the cannabinoid receptor CB1 in CGNs and HEK293 cells using the indicated $G_{i/o}$ protein sensors. Values are mean ± SEM from n independent experiments as indicated. Statistical significance of pEC_{50} and E_{max} are analysed using one-way analysis of variance (ANOVA) with a Dunnett's *post-hoc* multiple comparison test (compared with CP 55940 group). ****p < 0.0001; ***p < 0.001, **p < 0.01 and not significant (ns).

		pEC_{50} ± SEM (n)			E_{max} ± SEM (n)		
		CP 55940	Win 55,212-2	Bay 59-3074	CP 55940	Win 55,212-2	Bay 59-3074
CGNs	G_{i1}	8.18 ± 0.08 (4)	7.20 ± 0.17 (4) ***	6.47 ± 0.11 (4)****	100	104.8 ± 3.3 (4) ns	70.7 ± 1.1 (4)****
	G_{oA}	7.26 ± 0.13 (4)	6.40 ± 0.06 (4) ***	6.20 ± 0.08 (3) ***	100	130.7 ± 3.3 (4)****	42.1 ± 3.9 (3)****
HEK293 cells transfected CB1	G_{i1}	6.90 ± 0.13 (5)	6.45 ± 0.21 (5) ns	6.15 ± 0.18 (4) *	100	95.5 ± 3.1 (5) ns	50.3 ± 2.3 (4)****
	G_{oA}	6.78 ± 0.08 (5)	6.12 ± 0.11 (4 ****	5.93 ± 0.06 (4) ****	100	107.7 ± 9.6 (5) ns	56.3 ± 4.9 (4) ****

Supplementary Table 3. G protein response bias analysis of CP 55940 and Bay 59-3074 using Win 55,212-2 as reference (ref.) in CGNs and transfected HEK293 cells.

	G_{i1}					G_{oA}					Ligand bias		
	E_{max}	EC_{50}(M)	E_{max}/EC_{50}	$Log(E_{max}/EC_{50})$	$\triangle Log(E_{max}/EC_{50})$	E_{max}	EC_{50}(M)	E_{max}/EC_{50}	$Log(E_{max}/EC_{50})$	$\triangle Log(E_{max}/EC_{50})$	$\triangle\triangle log(E_{max}/EC_{50})$	Bias factor	For
**CGNs**													
Win 55,212-2	1.00	6.31E-08	1.58E+07	7.20	0.00	1.00	3.98E-07	2.51E+06	6.40	0.00	0.00	1	ref.
CP 55940	0.95	6.61E-09	1.44E+08	8.16	0.96	0.77	5.50E-08	1.39E+07	7.14	0.74	0.22	1.6	G_{i1}
Bay 59-3074	0.67	3.39E-07	1.99E+06	6.30	-0.90	0.32	6.31E-07	5.11E+05	5.71	-0.69	0.21	1.6	G_{oA}
**HEK293 cells**													
Win 55,212-2	1.00	3.55E-07	2.82E+06	6.45	0.00	1.00	7.59E-07	1.32E+06	6.12	0.00	0.00	1	ref.
CP 55940	1.05	1.26E-07	8.32E+06	6.92	0.47	0.93	1.66E-07	5.59E+06	6.75	0.63	0.16	1.4	G_{oA}
Bay 59-3074	0.53	7.08E-07	7.44E+05	5.87	-0.58	0.52	1.17E-06	4.45E+05	5.65	-0.47	0.11	1.3	G_{oA}

Supplementary Figures

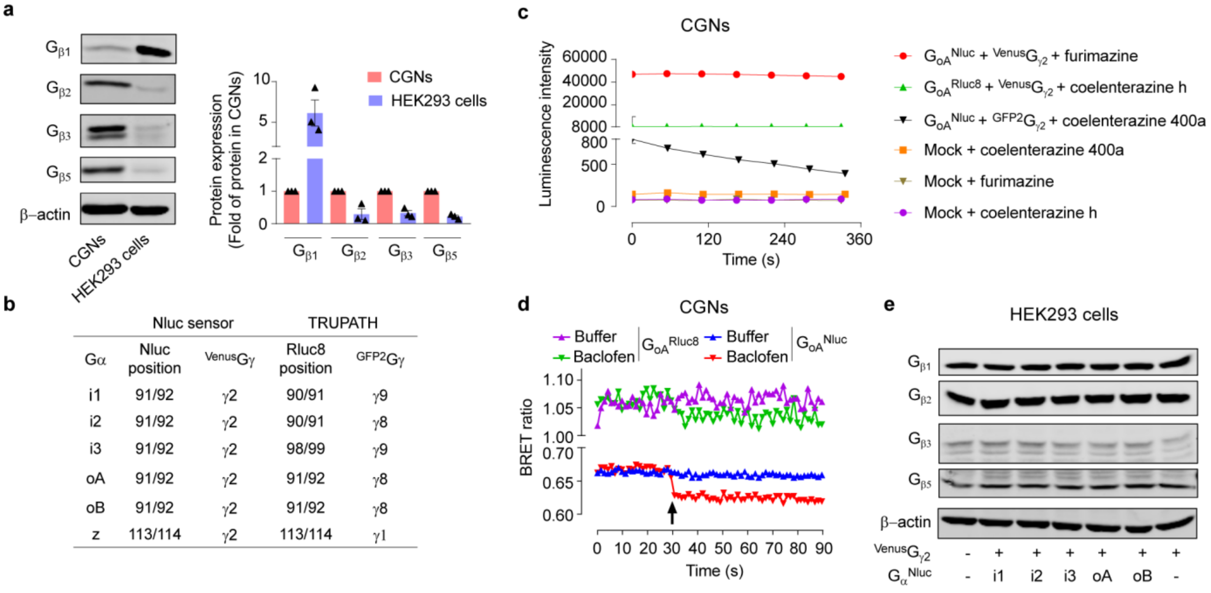

Supplementary Fig. 1. Validation of the $G_{i/o}$ sensors in CGNs. (a) Detection of endogenous $G\beta_1$, $G\beta_2$, $G\beta_3$ and $G\beta_5$ in CGNs and HEK293 cells by western blotting. Blots are representative from three independent experiments. Values are mean ± SEM from three biologically independent experiments. **(b)** Comparison of the constructs for the Nluc sensors developed in this study with the TRUPATH sensors for the indicated $G_{i/o}$ sensors. **(c)** Luminescence intensity measured in CGNs transfected with the indicated $G\alpha_{oA}$ luciferase constructs and $G\gamma_2$ fused with indicated fluorescence protein (50 ng each per well in 96-well plate). Coelenterazine 400a (10 µM) was added for $G_{oA}{}^{Rluc8}$ and ${}^{GFP2}G\gamma_2$ (TRUPATH sensor); coelenterazine H (5 µM) was added for $G_{oA}{}^{Rluc8}$ and ${}^{Venus}G\gamma_2$; furimazine (10 µM) was added for $G_{oA}{}^{Nluc}$ and ${}^{Venus}G\gamma_2$ in CGNs. For these three Go_A sensors, the signal was measured immediately after addition of these subtsrates. The effect of these three substrates was also measured in nontransfected CGNs (Mock). Data are representative from two independent experiments. **(d)** Kinetics of the BRET signal in CGNs co-expressing ${}^{Venus}G\gamma_2$ and $G\alpha_{oA}{}^{Nluc}$ or $G\alpha_{oA}{}^{Rluc8}$. Buffer or baclofen (100 µM) were injected at the indicated time (arrow). Data are representative from independent experiments (Nluc: n = 4; Rluc8: n = 2). **(e)** Comparison of the expression of endogenous $G\beta_1$, $G\beta_2$, $G\beta_3$ and $G\beta_5$ in HEK293 cells transfected with or without the indicated $G\alpha_{i1}{}^{Nluc}$ and ${}^{Venus}G\gamma_2$. Data are representative from three independent experiments. The raw data and p values are available in source data provided as a Source Data file.

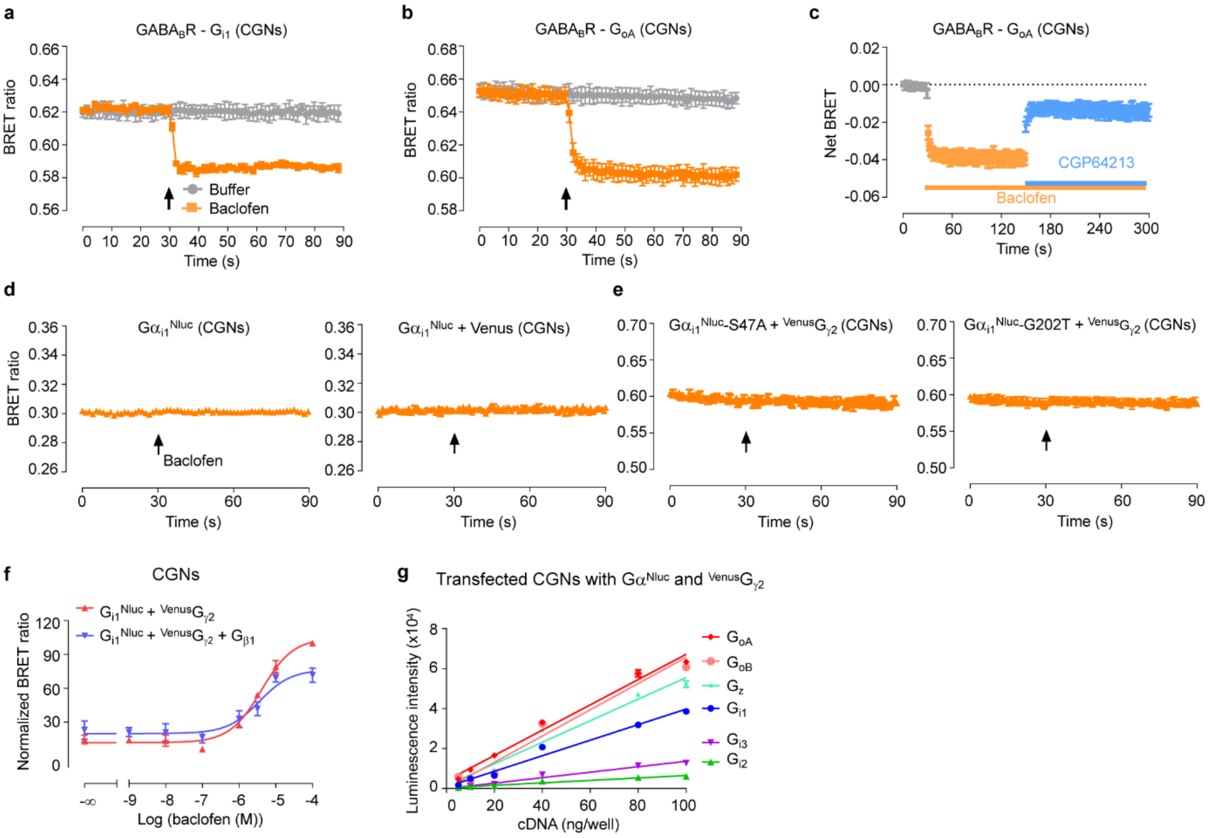

Supplementary Fig. 2. GABA$_B$ receptor-induced G$_{i/o}$ responses in CGNs. (a-c) Kinetics of the BRET signal between Gα^{Nluc} and ^{Venus}Gγ$_2$ in CGNs co-expressing ^{Venus}Gγ$_2$ and Gα$_{i1}$^{Nluc} or Gα$_{oA}$^{Nluc}. Buffer or baclofen (100 μM) or competitive antagonist CGP64213 (10 μM) were injected at the indicated time (arrow). **(d-e)** Kinetics of the BRET signal in CGNs expressing G$_{i1}$^{Nluc} alone, or G$_{i1}$^{Nluc} and Venus, or G$_{i1}$^{Nluc}-S47A and ^{Venus}G$_{\gamma 2}$, or G$_{i1}$^{Nluc}-G202T and ^{Venus}G$_{\gamma 2}$. Baclofen (100 μM) was injected at the indicated time (arrow). **(f)** Change of BRET signal between Gα$_{i1}$^{Nluc} and ^{Venus}Gγ$_2$ induced by baclofen, in CGNs co-expressing ^{Venus}Gγ$_2$ and Gα$_{i1}$^{Nluc} (50 ng each/well), or ^{Venus}Gγ$_2$, Gβ$_1$ and Gα$_{i1}$^{Nluc} (50 ng each/well). The data presented are as mean ± SEM of the BRET ratios from three independent experiments. **(g)** Effect of the quantity of transfected cDNA (5, 10, 20, 40, 80 and 100 ng/well in 96-well plate) for the constructs Gα$_{i1}$^{Nluc}, Gα$_{i2}$^{Nluc}, Gα$_{i3}$^{Nluc}, Gα$_{oA}$^{Nluc}, Gα$_{oB}$^{Nluc} or Gα$_z$^{Nluc}, together with ^{Venus}Gγ$_2$ (50 ng/well) on the luminescence signal of the luciferase at 480 nm in CGNs. Data are representative from at least three independent experiments in *(a-e, g)*. *(a)*, n = 5; *(b)*, n = 4; *(c)*, n = 4; *(d)*, n = 3; *(e)*, n = 3; *(g)*, n = 3. The raw data and p values are available in source data provided as a Source Data file.

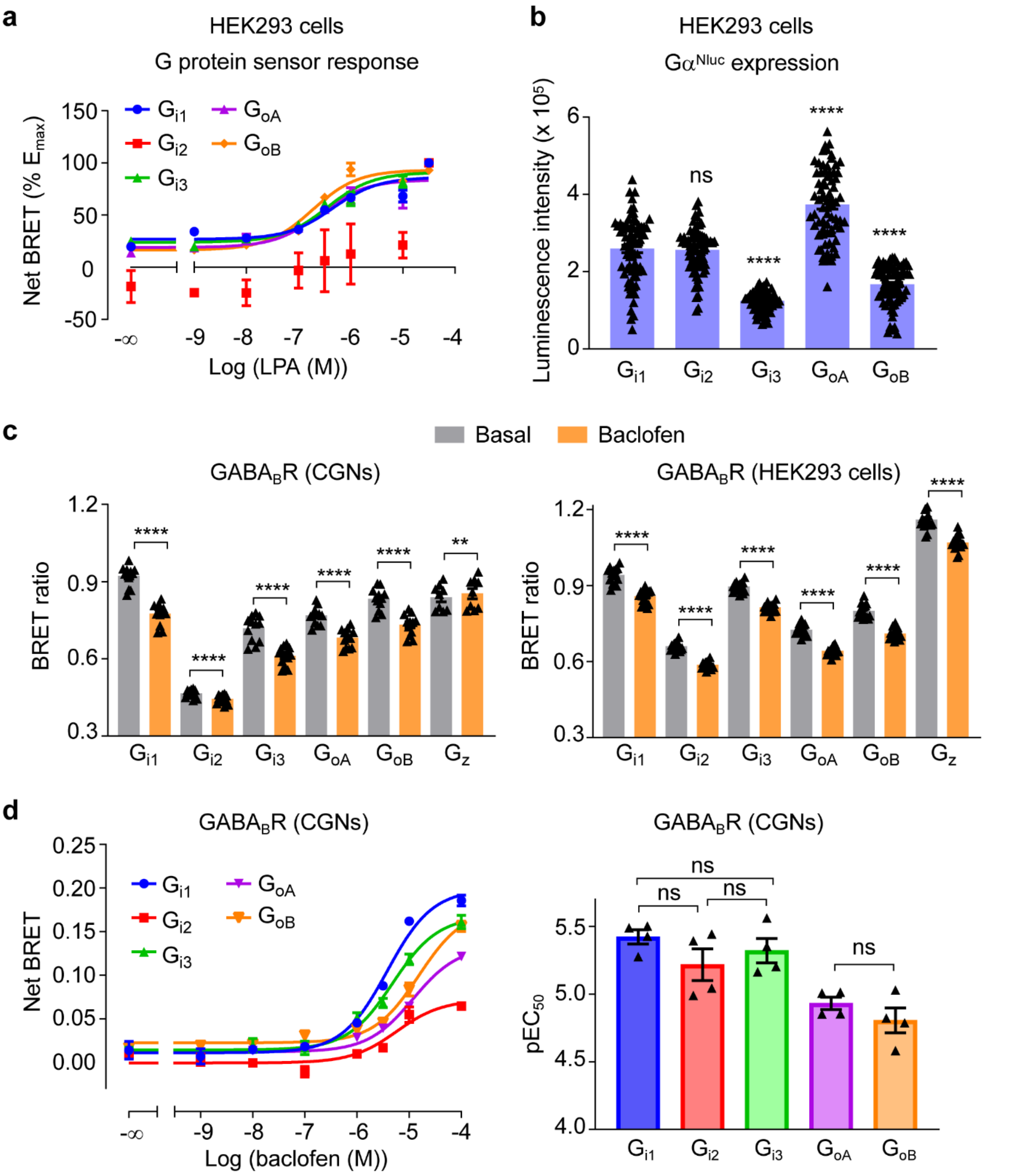

Supplementary Fig. 3. BRET signal and expression of the different $G_{i/o}$ protein sensors in HEK293 cells and CGNs. (a) Change of BRET signal upon different concentration of LPA treatment in HEK293 cells co-transfected with $^{Venus}G\gamma_2$ and the Nluc-tagged $G\alpha_{i1}$, $G\alpha_{i2}$, $G\alpha_{i3}$, $G\alpha_{oA}$ or $G\alpha_{oB}$. Values are mean ± SEM from three biologically independent experiments each performed in triplicate and normalized to maximum response. **(b)** Expression of the Nluc-tagged $G\alpha_{i1}$, $G\alpha_{i2}$, $G\alpha_{i3}$, $G\alpha_{oA}$ or $G\alpha_{oB}$ in *(a)*, measured by the luminescent signal at 480 nm. Values are mean ± SEM from three biologically independent experiments each performed in triplicate. The values of individual wells are shown. Data are analysed by using one-way analysis of variance (ANOVA) with a Dunnett's *post-hoc* multiple comparison test to determine significance (compared with G_{i1} group). ****$p < 0.0001$ and not significant (ns). **(c)** Change of BRET signal upon baclofen treatment in the cells in *Fig. 2a*: HEK293 cells co-transfected with GB1, GB2, $G\beta_1$, $^{Venus}G\gamma_2$ and

the Nluc-tagged $G\alpha_{i1}$, $G\alpha_{i2}$, $G\alpha_{i3}$, $G\alpha_{oA}$, $G\alpha_{oB}$ or $G\alpha_{z}$; CGNs co-transfected with $^{Venus}G\gamma_2$ and the Nluc-tagged $G\alpha_{i1}$, $G\alpha_{i2}$, $G\alpha_{i3}$, $G\alpha_{oA}$, $G\alpha_{oB}$ or $G\alpha_{z}$. Values are mean ± SEM from four biologically independent experiments each performed triplicate or quadruplicate. The values of individual wells are shown. Data are analysed by using a paired *t*-test between basal and baclofen treatment. ****p < 0.0001 and **p < 0.01. **(d)** Dose-response and pEC_{50} of baclofen-induced BRET change between the indicated $G\alpha_{i/o}{}^{Nluc}$ and $^{Venus}G\gamma_2$ in CGNs (n = 4). Data are analysed by using one-way analysis of variance (ANOVA) with a Dunnett's *post-hoc* multiple comparison test to determine significance. not significant (ns). The raw data and p values are available in source data provided as a Source Data file.

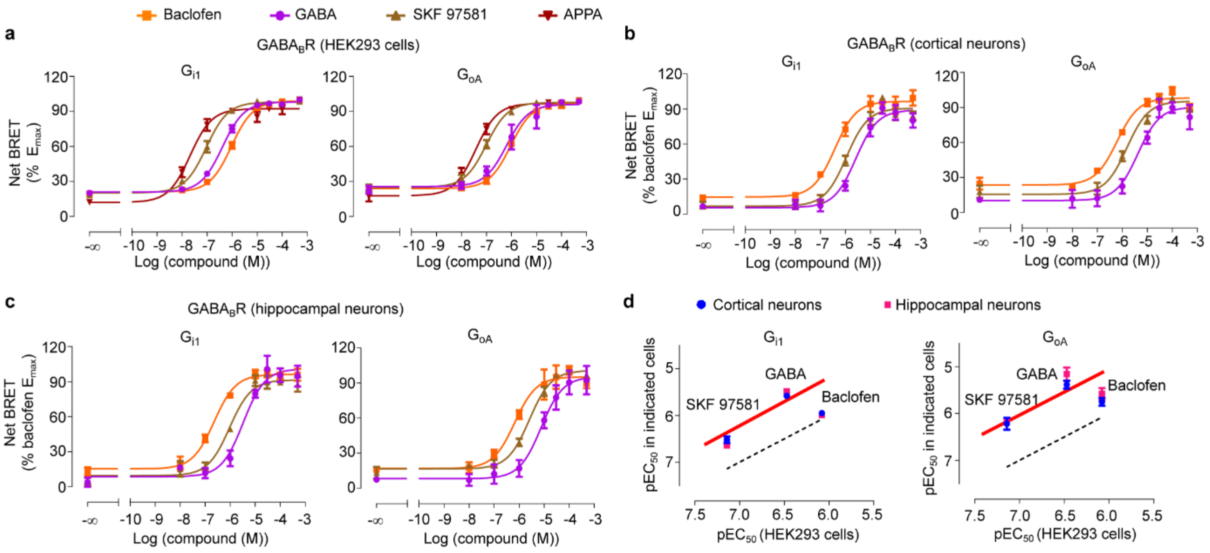

Supplementary Fig. 4. Ligand potencies of the endogenous $GABA_B$ receptor in cortical and hippocampal neurons compared to HEK293 cells. (a) Change of BRET signal between $G\alpha^{Nluc}$ and $^{Venus}G\gamma_2$ induced by the indicated agonists of the $GABA_B$ receptor in HEK293 cells co-transfected with GB1, GB2, $G\beta_1$, $^{Venus}G\gamma_2$ and $G\alpha_{i1}{}^{Nluc}$ or $G\alpha_{oA}{}^{Nluc}$. **(b-c)** Change of BRET signal between $G\alpha^{Nluc}$ and $^{Venus}G\gamma_2$ induced by the indicated agonists of the $GABA_B$ receptor in cortical neurons or hippocampal neurons co-transfected with $^{Venus}G\gamma_2$ and $G\alpha_{i1}{}^{Nluc}$ or $G\alpha_{oA}{}^{Nluc}$. **(d)** Correlation of the agonist potencies (pEC_{50}) between HEK293 cells and cortical neurons or hippocampal neurons determined by the G_{i1} and G_{oA} BRET sensors in *(a-c)*. Dotted lines are the correlation of pEC_{50} determined with HEK293 cells. Red lines are the fit of pEC_{50} in cortical neurons or hippocampal neurons with the same slope as dotted lines. Data are mean ± SEM from at least three biologically independent experiments each performed in triplicate, in *a-c*. *(a),* baclofen, GABA and SKF 97581, n = 4; APPA, n = 3. *(b),* G_{i1}, n = 4; G_{oA}, n = 3. *(c),* G_{i1}, n = 3; G_{oA}, n = 3. Data are normalized to maximum baclofen response in *(a, b, c)*. The raw data and p values are available in source data provided as a Source Data file.

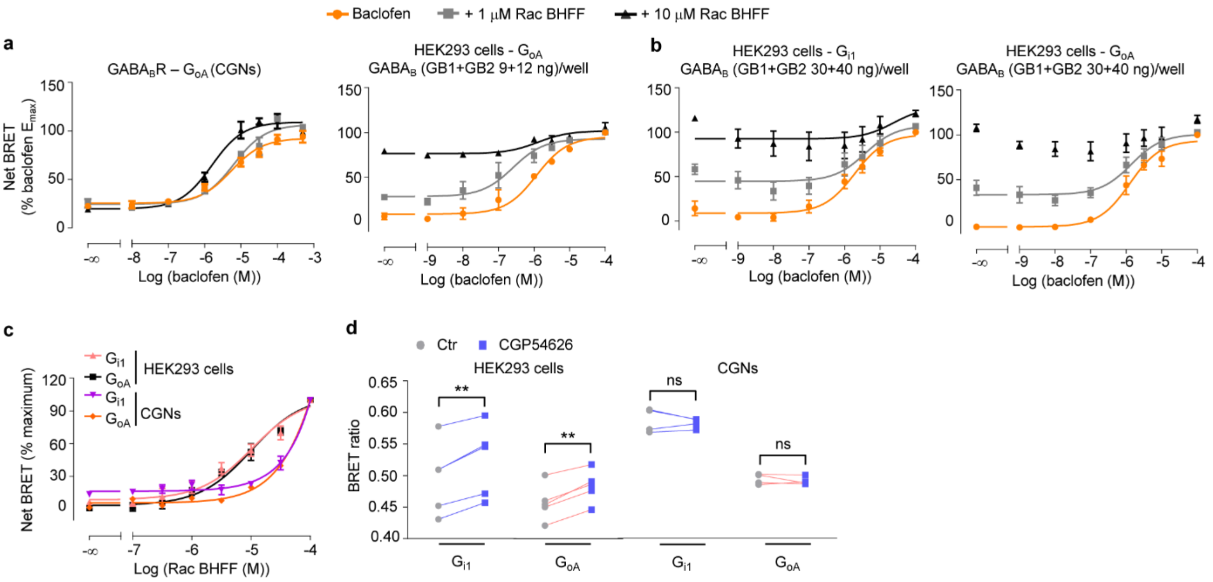

Supplementary Fig. 5. Effect of PAM Rac BHFF and antagonist CGP54626 on the change in GABA$_B$ receptor activity in CGNs and HEK293 cells. (a) The effect of Rac BHFF in baclofen-induced G_{oA} response in transfected CGNs and HEK293 cells (GB1 + GB2: 9 ng + 12 ng per well for 96-well plate). **(b)** The effect of Rac BHFF in baclofen-induced G_{i1} and G_{oA} response in transfected HEK293 cells (GB1 + GB2: 30 ng + 40 ng per well for 96-well plate). **(c)** The effect of Rac BHFF alone in G_{i1} and G_{oA} response in transfected HEK293 cells and CGNs. Data are mean ± SEM from three biologically independent experiments each performed in triplicate, in *a-c*. Data are normalized to maximum baclofen response in *(a, b)* and normalized to Rac BHFF maximum response in *(c)*. **(d)** BRET ratio of the G_{i1} and G_{oA} sensors in absence (control, Ctr) or presence of CGP54626 (50 μM for 30 min) in CGNs co-expressing $^{Venus}G\gamma_2$ and $G\alpha_{i1}{}^{Nluc}$ or $G\alpha_{oA}{}^{Nluc}$ and in HEK293 cells co-transfected with GB1, GB2, $G\beta_1$, $^{Venus}G\gamma_2$ and $G\alpha_{i1}{}^{Nluc}$ or $G\alpha_{oA}{}^{Nluc}$. Data are from biologically independent experiments (CGNs, n = 4; HEK293 cells, n = 5), and analysed using a paired-t test. **$p < 0.01$ and not significant (ns). The raw data and p values are available in source data provided as a Source Data file.

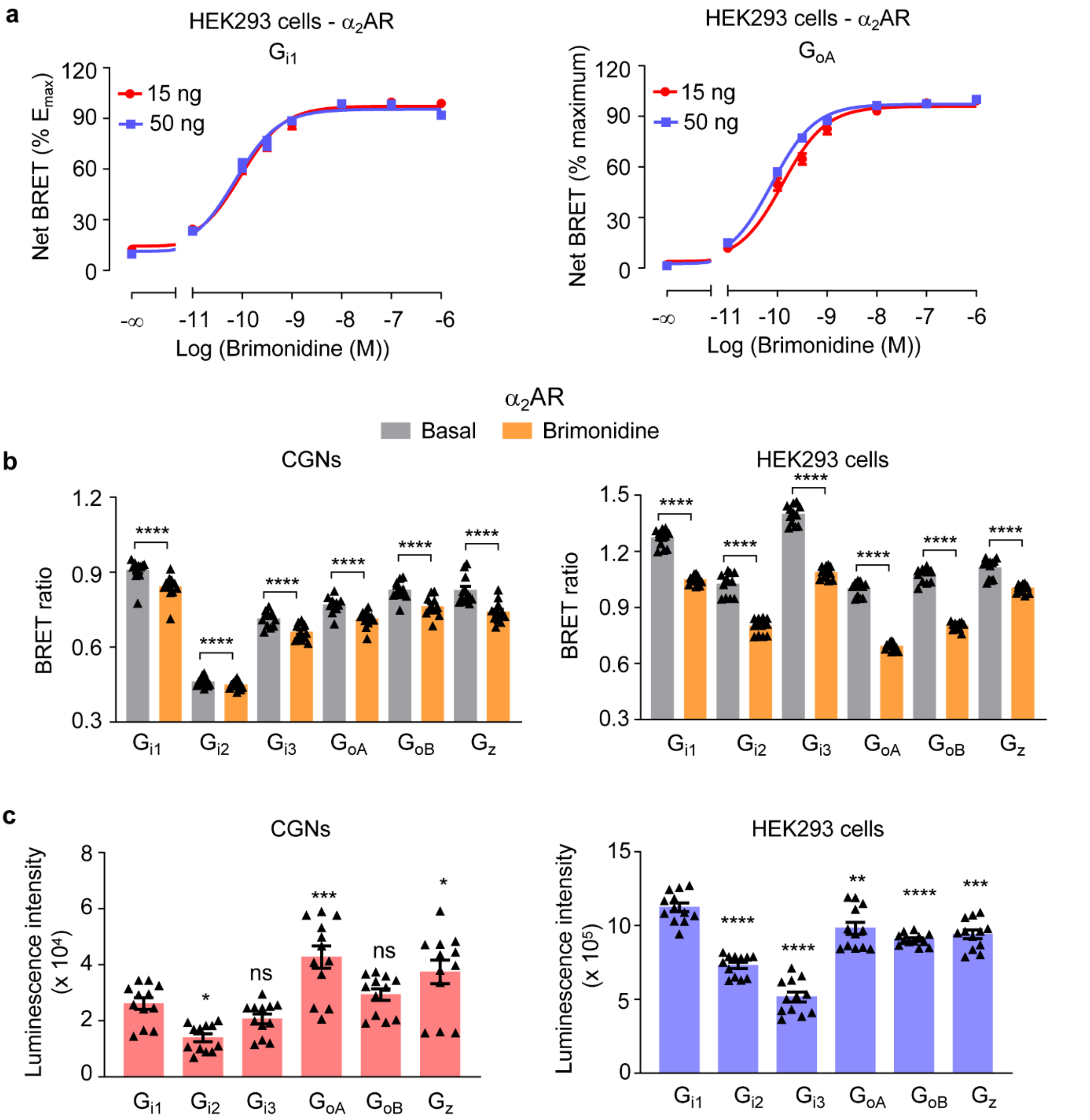

Supplementary Fig. 6. Change of BRET signal induced by α_2AR and expression of the different $G_{i/o}$ protein sensors in CGNs and HEK293 cells. (a) Dose-response of brimonidine-induced BRET change for the indicated G_{i1} or G_{oA} sensors in HEK293 cells co-transfected with mouse α_{2A}AR (15 ng or 50 ng), $G\beta_1$, $^{Venus}G\gamma_2$ and $G\alpha_{i1}{}^{Nluc}$ or $G\alpha_{oA}{}^{Nluc}$, for the indicated amount of α_{2A}AR cDNA. Values are mean ± SEM from four biologically independent experiments each performed in triplicate. **(b)** Change of BRET signal upon brimonidine treatment in the cells in *Fig. 5e* (CGNs co-transfected with $^{Venus}G\gamma_2$ and the Nluc-tagged $G\alpha_{i1}$, $G\alpha_{i2}$, $G\alpha_{i3}$, $G\alpha_{oA}$, $G\alpha_{oB}$ or $G\alpha_z$; HEK293 cells co-transfected with mouse α_{2A}AR, $G\beta_1$, $^{Venus}G\gamma_2$ and the Nluc-tagged $G\alpha_{i1}$, $G\alpha_{i2}$, $G\alpha_{i3}$, $G\alpha_{oA}$, $G\alpha_{oB}$ or $G\alpha_z$). Values are mean ± SEM from four biologically independent experiments each performed in triplicate or quadruplicate. Data are analysed by using a paired *t*-test between basal and brimonidine treatment. ****$p < 0.0001$. **(c)** Expression of the Nluc-tagged $G\alpha_{i1}$, $G\alpha_{i2}$, $G\alpha_{i3}$, $G\alpha_{oA}$, $G\alpha_{oB}$ or $G\alpha_z$ in transfected CGNs or HEK293 cells in *Fig. 5e*, measured by the luminescent signal at 480 nm. Values are mean ± SEM from four biologically independent experiments each performed in triplicate or quadruplicate. The values of individual wells are shown. Data are analysed by using one-way analysis of variance (ANOVA) with a Dunnett's *post-hoc* multiple comparison test to

determine significance (compared with G_{i1} group). ***$p < 0.001$, *$p < 0.05$ and not significant (ns). The raw data and p values are available in source data provided as a Source Data file.

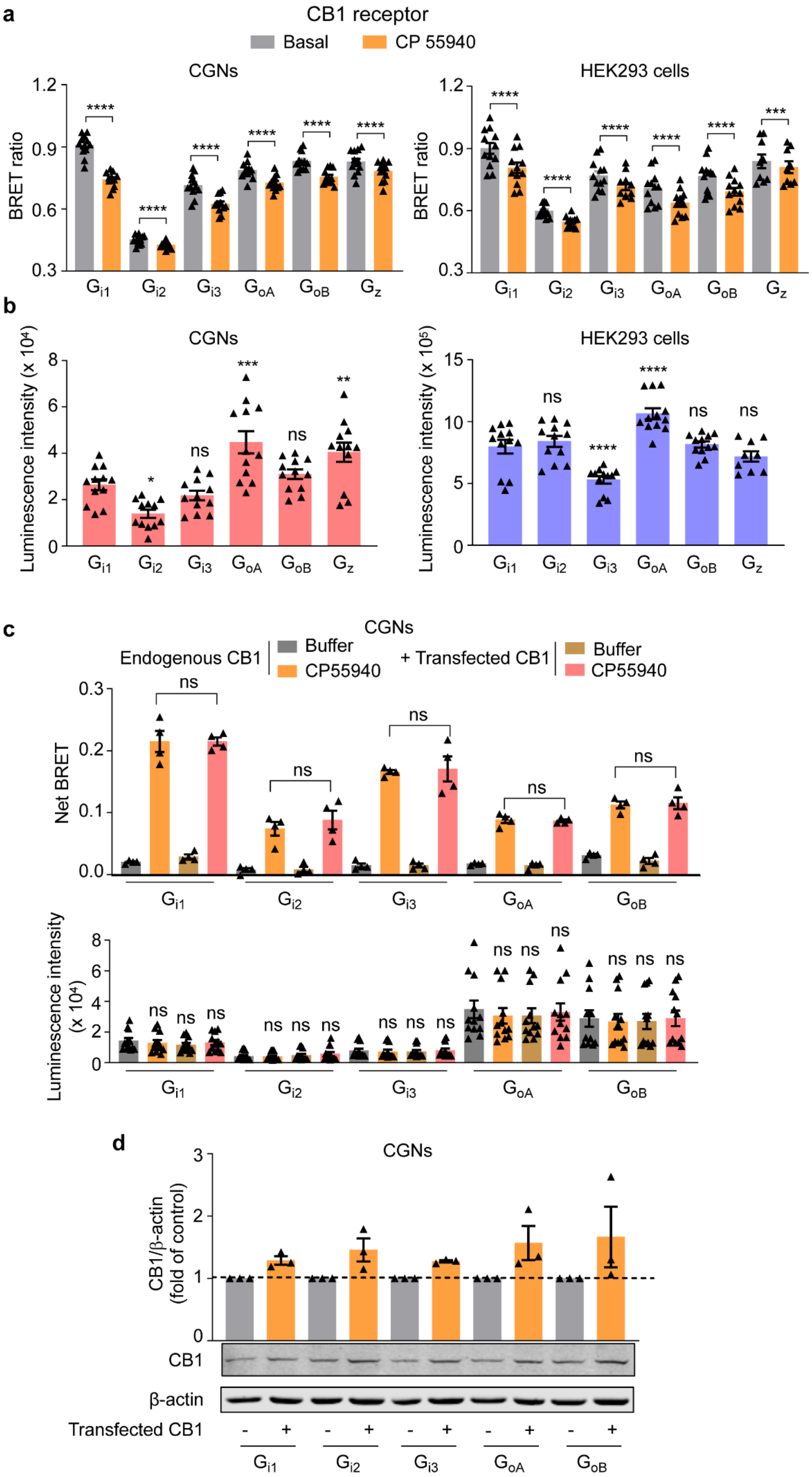

a
CB1 receptor
Basal
CP 55940
CGNs
HEK293 cells
BRET ratio
b
Luminescence intensity (x 10⁴)
Luminescence intensity (x 10⁵)
c
CGNs
Endogenous CB1
Buffer
CP55940
+ Transfected CB1
Net BRET
Luminescence intensity (x 10⁴)
d
CGNs
CB1/β-actin (fold of control)
CB1
β-actin
Transfected CB1

Supplementary Fig. 7. Change of BRET signal induced by CB1 receptor and expression of the different $G_{i/o}$ protein sensors in CGNs and HEK293 cells. (a) Change of BRET signal upon CP 55940 treatment in the cells in *Fig. 6b* (CGNs co-transfected with $^{Venus}G\gamma_2$ and the Nluc-tagged $G\alpha_{i1}$, $G\alpha_{i2}$, $G\alpha_{i3}$, $G\alpha_{oA}$, $G\alpha_{oB}$ or $G\alpha_z$; HEK293 cells co-transfected with mouse CB1, $G\beta_1$, $^{Venus}G\gamma_2$ and the Nluc-tagged $G\alpha_{i1}$, $G\alpha_{i2}$, $G\alpha_{i3}$, $G\alpha_{oA}$, $G\alpha_{oB}$ or $G\alpha_z$). Values are mean ± SEM from four biologically independent experiments each performed in triplicate. Data are analysed by using a paired *t*-test between basal and CP 55940 treatment. ****$p < 0.0001$ and ***$p < 0.001$. **(b)** Expression of the Nluc-tagged $G\alpha_{i1}$, $G\alpha_{i2}$, $G\alpha_{i3}$, $G\alpha_{oA}$, $G\alpha_{oB}$ or $G\alpha_z$ in transfected CGNs and HEK293 cells in *Fig. 6b*, measured by the luminescent signal at 480 nm. Values are mean ± SEM from four biologically independent experiments each performed in triplicate. The values of individual well are shown. Data are analysed by using one-way analysis of variance (ANOVA) with a Dunnett's *post-hoc* multiple comparison test to determine significance (compared with G_{i1} group). ****$p < 0.0001$, ***$p < 0.001$, **$p < 0.01$, *$p < 0.05$ and not significant (ns). **(c)** CP 55940-induced net BRET between $G\alpha^{Nluc}$ and $^{Venus}G\gamma$ in CGNs with or without CB1 transfection (50 ng/well), for the indicated $G_{i/o}$ sensors. CGNs were co-transfected only with indicated $^{Venus}G\gamma_2$ and the Nluc-tagged $G\alpha_{i1}$, $G\alpha_{i2}$, $G\alpha_{i3}$, $G\alpha_{oA}$ or $G\alpha_{oB}$. Values are mean ± SEM from four biologically independent experiments each performed in triplicate. The expression of the indicated Nluc-tagged $G\alpha_{i/o}$ in these CGNs was measured by the luminescent signal at 480 nm. Values are mean ± SEM from three biologically independent experiments each performed in triplicate. The values of individual well are shown. **(d)** Western blotting detection of CB1 and β-actin in the CGNs in *(c)*. The raw data and p values are available in source data provided as a Source Data file.

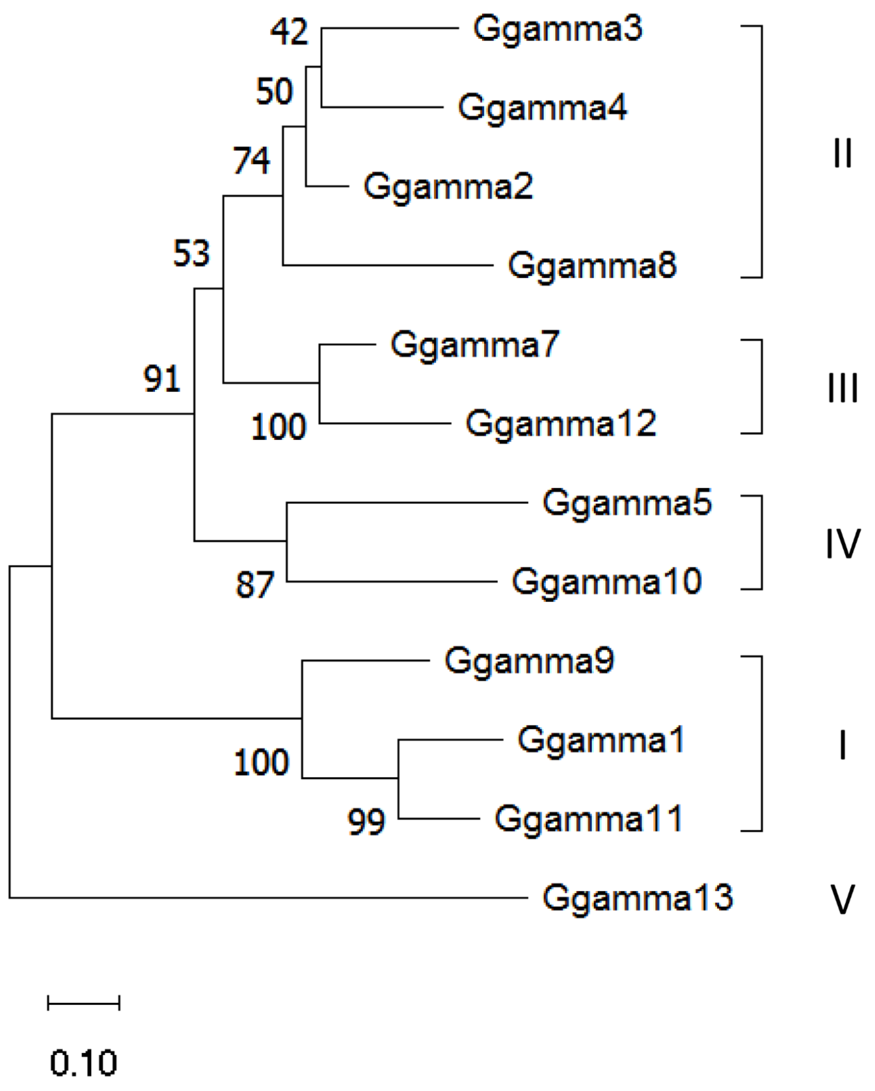

Supplementary Fig. 8. Phylogenetic trees of human Gγ subunits. Gγ subunits are classified in I-V groups using MegaX software. The numbers at the nodes of the branches represent the percentage of the bootstrap test (1000 replicates). Scale bar, 0.1 = 10% genetic distance.

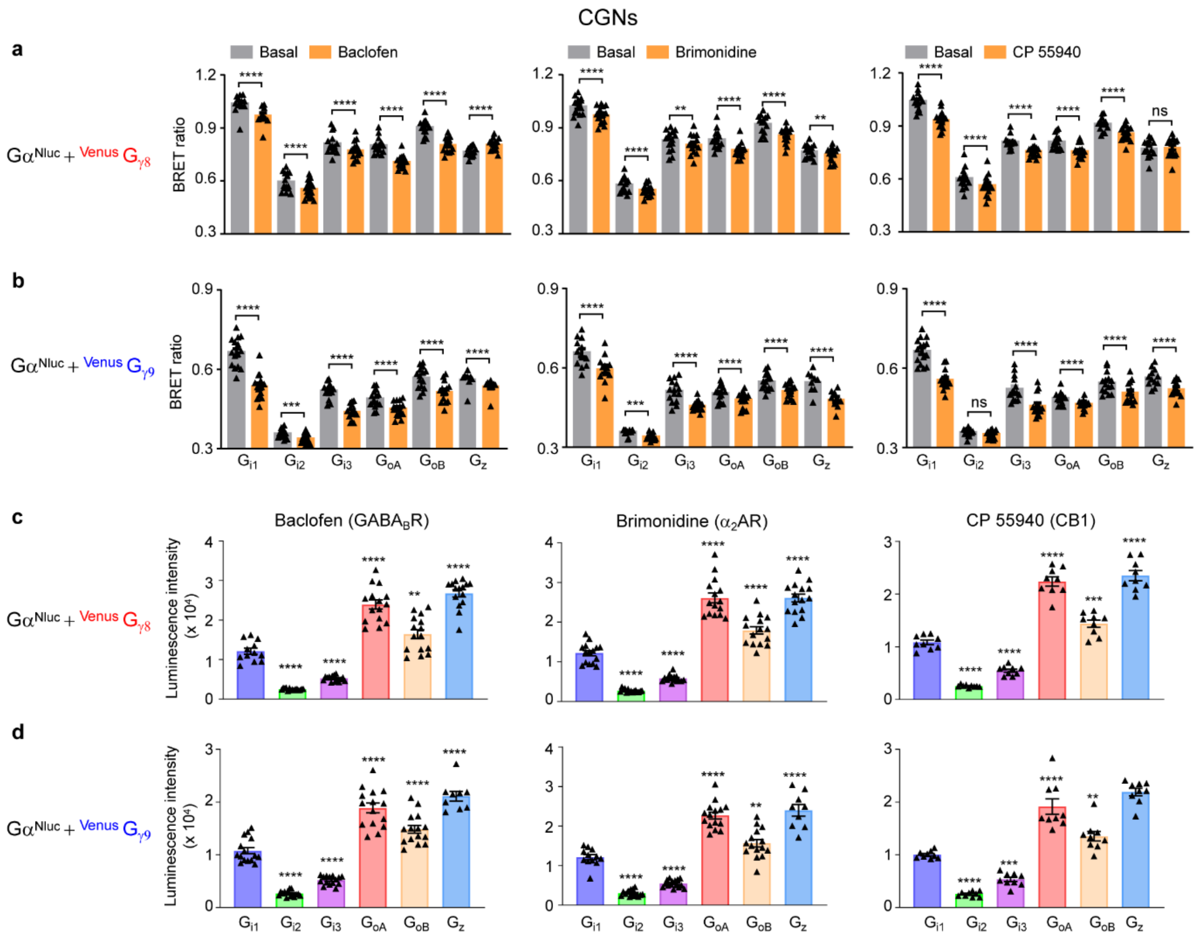

Supplementary Fig. 9. Change of BRET signal induced by the indicated receptors and expression of the different $G_{i/o}$ protein sensors in CGNs. (a-b) Change of BRET signal upon baclofen, brimonidine or CP 55940 treatment in CGNs co-transfected with $^{Venus}G\gamma_8$ or $^{Venus}G\gamma_9$ together with the Nluc-tagged $G\alpha_{i1}$, $G\alpha_{i2}$, $G\alpha_{i3}$, $G\alpha_{oA}$, $G\alpha_{oB}$ or $G\alpha_z$ (referred to *Fig. 7a-b*). Values are mean ± SEM from biologically independent experiments (baclofen and brimonidine, n = 5; CP 55940, n = 3) each performed in triplicate. Data are analysed by using a paired *t*-test between basal and indicated drug treatment. ****$p < 0.0001$, ***$p < 0.001$, **$p < 0.01$ and not significant (ns). **(c-d)** Expression of the Nluc-tagged $G\alpha_{i1}$, $G\alpha_{i2}$, $G\alpha_{i3}$, $G\alpha_{oA}$, $G\alpha_{oB}$ or $G\alpha_z$ in transfected CGNs in *Fig. 7a-b*, measured by the luminescent signal at 480 nm. Values are mean ± SEM from biologically independent experiments (baclofen and brimonidine, n = 5; CP 55940, n = 3) each performed in triplicate. The values of individual well are shown. Data are analysed by using one-way analysis of variance (ANOVA) with a Dunnett's *post-hoc* multiple comparison test to determine significance (compared with G_{i1} group). ****$p < 0.0001$, ***$p < 0.001$ and **$p < 0.01$. The raw data and p values are available in source data provided as a Source Data file.

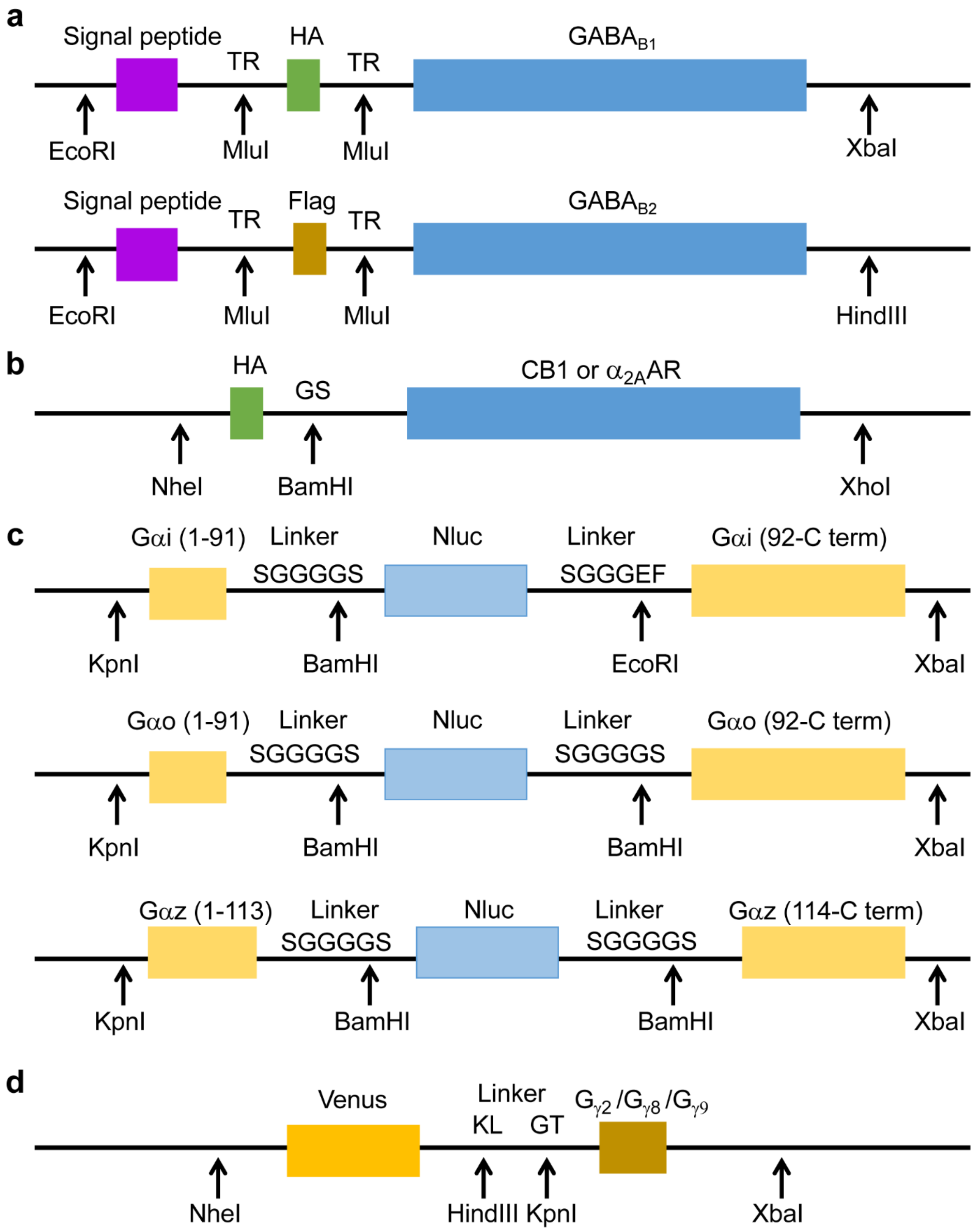

Supplementary Fig. 10. Schematic representation of the constructs used **(a)** for GABA$_B$ receptor (GABA$_{B1}$ and GABA$_{B2}$ subunits), **(b)** for CB1 or α$_{2a}$AR receptors, **(c)** for Gα$_{i1}^{Nluc}$, Gα$_{i2}^{Nluc}$, Gα$_{i3}^{Nluc}$, Gα$_{oA}^{Nluc}$, Gα$_{oB}^{Nluc}$ and Gα$_{z}^{Nluc}$, **(d)** for ^{Venus}Gγ$_2$, ^{Venus}Gγ$_8$ and ^{Venus}Gγ$_9$. TR, GS, SGGGS, SGGGEF, KLGT are amino acid linkers.

Rat HA-GB1
MVLLLILSVLLLKEDVRGSAQSTRYPYDVPDYATRGGAQTPNATSEGCQIIHPPWEGGIRYRGLTRDQVKAI
NFLPVDYEIEYVCRGEREVVGPKVRKCLANGSWTDMDTPSRCVRICSKSYLTLENGKVFLTGGDLPALDGA
RVEFRCDPDFHLVGSSRSVCSQGQWSTPKPHCQVNRTPHSERRAVYIGALFPMSGGWPGGQACQPAVEMAL
EDVNSRRDILPDYELKLIHHDSKCDPGQATKYLYELLYNDPIKIILMPGCSSVSTLVAEAARMWNLIVLSYGS
SSPALSNRQRFPTFFRTHPSATLHNPTRVKLFEKWGWKKIATIQQTTEVFTSTLDDLEERVKEAGIEITFRQSFF
SDPAVPVKNLKRQDARIIVGLFYETEARKVFCEVYKERLFGKKYVWFLIGWYADNWFKTYDPSINCTVEEM
TEAVEGHITTEIVMLNPANTRSISNMTSQEFVEKLTKRLKRHPEETGGFQEAPLAYDAIWALALALNKTSGG
GGRSGVRLEDFNYNNQTITDQIYRAMNSSSFEGVSGHVVFDASGSRMAWTLIEQLQGGSYKKIGYYDSTKD
DLSWSKTDKWIGGSPPADQTLVIKTFRFLSQKLFISVSVLSSLGIVLAVVCLSFNIYNSHVRYIQNSQPNLNNL
TAVGCSLALAAVFPLGLDGYHIGRSQFPFVCQARLWLLGLGFSLGYGSMFTKIWWVHTVFTKKEEKKEWR
KTLEPWKLYATVGLLVGMDVLTLAIWQIVDPLHRTIETFAKEEPKEDIDVSILPQLEHCSSKKMNTWLGIFYG
YKGLLLLLGIFLAYETKSVSTEKINDHRAVGMAIYNVAVLCLITAPVTMILSSQQDAAFAFASLAIVFSSYITLV
VLFVPKMRRLITRGEWQSETQDTMKTGSSTNNNEEEKSRLLEKENRELEKIIAEKEERVSELRHQLQSRQQL
RSRRHPPTPPDPSGGLPRGPSEPPDRLSCDGSRVHLLYK*

Rat Flag-GB2
MVLLLILSVLLLKEDVRGSAQSTRPVDYKDDDDKTRWTRGAPRPPPSSPPLSIMGLMPLTKEVAKGSIGRGV
LPAVELAIEQIRNESLLRPYFLDLRLYDTECDNAKGLKAFYDAIKYGPNHLMVFGGVCPSVTSIIAESLQGWN
LVQLSFAATTPVLADKKKYPYFFRTVPSDNAVNPAILKLLKHFRWRRVGTLTQDVQRFSEVRNDLTGVLYGE
DIEISDTESFSNDPCTSVKKLKGNDVRIILGQFDQNMAAKVFCCAFEESMFGSKYQWIIPGWYEPAWWEQV
HVEANSSRCLRRSLLAAMEGYIGVDFEPLSSKQIKTISGKTPQQYEREYNTKRSGVGPSKFHGYAYDGIWVI
AKTLQRAMETLHASSRHQRIQDFNYTDHTLGKIILNAMNETNFFGVTGQVVFRNGERMGTIKFTQFQDSRE
VKVGEYNAVADTLEIINDTIRFQGSEPPKDKTIILEQLRKISLPLYSILSALTILGMIMASAFLFFNIKNRNQKLI
KMSSPYMNNLIILGGMLSYASIFLFGLDGSFVSEKTFETLCTVRTWILTVGYTTAFGAMFAKTWRVHAIFKN
VKMKKKIIKDQKLLVIVGGMLLIDLCILICWQAVDPLRRTVERYSMEPDPAGRDISIRPLLEHCENTHMTIWL
GIVYAYKGLLMLFGCFLAWETRNVSIPALNDSKYIGMSVYNVGIMCIIGAAVSFLTRDQPNVQFCIVALVIIFC
STITLCLVFVPKLITLRTNPDAATQNRRFQFTQNQKKEDSKTSTSVTSVNQASTSRLEGLQSENHRLRMKITE
LDKDLEEVTMQLQDTPEKTTYIKQNHYQELNDILSLGNFTESTDGGKAILKNHLDQNPQLQWNTTEPSRTC
KDPIEDINSPEHIQRRLSLQLPILHHAYLPSIGGVDASCVSPCVSPTASPRHRHVPPSFRVMVSGL*

Mouse HA-α_{2A}AR
MYPYDVPDYAGSFRQEQPLAEGSFAPMGSLQPDAGNSSWNGTEAPGGGTRATPYSLQVTLTLVCLAGLLM
LFTVFGNVLVIIAVFTSRALKAPQNLFLVSLASADILVATLVIPFSLANEVMGYWYFGKVWCEIYLALDVLFC
TSSIVHLCAISLDRYWSITQAIEYNLKRTPRRIKAIIVTVWVISAVISFPPLISIEKKGAGGGQQPAEPSCKINDQ
KWYVISSSIGSFFAPCLIMILVYVRIYQIAKRRTRVPPSRRGPDACSAPPGGADRRPNGLGPERGAGPTGAEAE
PLPTQLNGAPGEPAPAGPRDGDALDLEESSSSEHAERPPGPRRPDRGPRAKGKTRASQVKPGDSLPRRGPGA
AGPGASGSGHGEERGGGAKASRWRGRQNREKRFTFVLAVVIGVFVVCWFPFFFTYTLIAVGCPVPSQLFNFF
FWFGYCNSSLNPVIYTIFNHDFRRAFKKILCRGDRKRIV*

Mouse HA-CB1
MYPYDVPDYAGSKSILDGLADTTFRTITTDLLYVGSNDIQYEDIKGDMASKLGYFPQKFPLTSFRGSPFQEK
MTAGDNSPLVPAGDTTNITEFYNKSLSSFKENEDNIQCGENFMDMECFMILNPSQQLAIAVLSLTLGTFTVLE
NLLVLCVILHSRSLRCRPSYHFIGSLAVADLLGSVIFVYSFVDFHVFHRKDSPNVFLFKLGGVTASFTASVGSL
FLTAIDRYISIHRPLAYKRIVTRPKAVVAFCLMWTIAIVIAVLPLLGWNCKKLQSVCSDIFPLIDETYLMFWIGV
TSVLLLFIVYAYMYILWKAHSHAVRMIQRGTQKSIIIHTSEDGKVQVTRPDQARMDIRLAKTLVLILVVLIICW
GPLLAIMVYDVFGKMNKLIKTVFAFCSMLCLLNSTVNPIIYALRSKDLRHAFRSMFPSCEGTAQPLDNSMGD
SDCLHKHANNTASMHRAAESCIKSTVKIAKVTMSVSTDTSAEAL*

Supplementary Fig. 11. Sequences of the constructs used for the rat $GABA_B$ receptor (GB1 and GB2 subunits), mouse α_{2A}AR and mouse CB1 receptor. The amino acid sequence of the signal peptide of GB1 and GB2 (dark red), HA tag of GB1, α_{2A}AR and CB1 (red), Flag tag of GB2 (blue), amino acid linkers (green) and receptor (black) are indicated.

$G_{\alpha i1}^{Nluc}$

MGCTLSAEDKAAVERSKMIDRNLREDGEKAAREVKLLLLGAGESGKSTIVKQMKIIHEAGYSEEECKQYKAVVYSNTIQSIIAIIRAM
GRLSGGGGSVFTLEDFVGDWRQTAGYNLDQVLEQGGVSSLFQNLGVSVTPIQRIVLSGENGLKIDIHVIIPYEGLSGDQMGQIEKIFK
VVYPVDDHHFKVILHYGTLVIDGVTPNMIDYFGRPYEGIAVFDGKKITVTGTLWNGNKIIDERLINPDGSLLFRVTINGVTGWRLCERI
LASGGGEFKIDFGDSARADDARQLFVLAGAAEEGFMTAELAGVIKRLWKDSGVQACFNRSREYQLNDSAAYYLNDLDRIAQPNYIP
TQQDVLRTRVKTTGIVETHFTFKDLHFKMFDVGGQRSERKKWIHCFEGVTAIIFCVALSDYDLVLAEDEEMNRMHESMKLFDSICNN
KWFTDTSIILFLNKKDLFEEKIKKSPLTICYPEYAGSNTYEEAAAYIQCQFEDLNKRKDTKEIYTHFTCATDTKNVQFVFDAVTDVIIKN
NLKDCGLF*

$G_{\alpha i2}^{Nluc}$

MGCTVSAEDKAAAERSKMIDKNLREDGEKAAREVKLLLLGAGESGKSTIVKQMKIIHEDGYSEEECRQYRAVVYSNTIQSIMAIVK
AMGNLSGGGGSVFTLEDFVGDWRQTAGYNLDQVLEQGGVSSLFQNLGVSVTPIQRIVLSGENGLKIDIHVIIPYEGLSGDQMGQIEKI
FKVVYPVDDHHFKVILHYGTLVIDGVTPNMIDYFGRPYEGIAVFDGKKITVTGTLWNGNKIIDERLINPDGSLLFRVTINGVTGWRLC
ERILASGGGEFQIDFADPSRADDARQLFALSCTAEEQGVLPDDLSGVIRRLWADHGVQACFGRSREYQLNDSAAYYLNDLERIAQSD
YIPTQQDVLRTRVKTTGIVETHFTFKDLHFKMFDVGGQRSERKKWIHCFEGVTAIIFCVALSAYDLVLAEDEEMNRMHESMKLFDSIC
NNKWFTDTSIILFLNKKDLFEEKITHSPLTICFPEYTGANKYDEAASYIQSKFEDLNKRKDTKEIYTHFTCATDTKNVQFVFDAVTDVII
KNNLKDCGLF*

$G_{\alpha i3}^{Nluc}$

MGCTLSAEDKAAVERSKMIDRNLREDGEKAAKEVKLLLLGAGESGKSTIVKQMKIIHEDGYSEDECKQYKVVVYSNTIQSIIAIIRA
MGRLSGGGGSVFTLEDFVGDWRQTAGYNLDQVLEQGGVSSLFQNLGVSVTPIQRIVLSGENGLKIDIHVIIPYEGLSGDQMGQIEKIF
KVVYPVDDHHFKVILHYGTLVIDGVTPNMIDYFGRPYEGIAVFDGKKITVTGTLWNGNKIIDERLINPDGSLLFRVTINGVTGWRLCE
RILASGGGEFKIDFGEAARADDARQLFVLAGSAEEGVMTPELAGVIKRLWRDGGVQACFSRSREYQLNDSASYYLNDLDRISQSNYI
PTQQDVLRTRVKTTGIVETHFTFKDLYFKMFDVGGQRSERKKWIHCFEGVTAIIFCVALSDYDLVLAEDEEMNRMHESMKLFDSICN
NKWFTETSIILFLNKKDLFEEKIKRSPLTICYPEYTGSNTYEEAAAYIQCQFEDLNRRKDTKEIYTHFTCATDTKNVQFVFDAVTDVIIK
NNLKECGLY*

$G_{\alpha oA}^{Nluc}$

MGCTLSAEERAALERSKAIEKNLKEDGISAAKDVKLLLLGAGESGKSTIVKQMKIIHEDGFSGEDVKQYKPVVYSNTIQSLAAIVRA
MDTLSGGGGSVFTLEDFVGDWRQTAGYNLDQVLEQGGVSSLFQNLGVSVTPIQRIVLSGENGLKIDIHVIIPYEGLSGDQMGQIEKIF
KVVYPVDDHHFKVILHYGTLVIDGVTPNMIDYFGRPYEGIAVFDGKKITVTGTLWNGNKIIDERLINPDGSLLFRVTINGVTGWRLCE
RILASGGGGSGIEYGDKERKADAKMVCDVVSRMEDTEPFSAELLSAMMRLWGDSGIQECFNRSREYQLNDSAKYYLDSLDRIGAA
DYQPTEQDILRTRVKTTGIVETHFTFKNLHFRLFDVGGQRSERKKWIHCFEDVTAIIFCVALSGYDQVLHEDETTNRMHESLMLFDSI
CNNKFFIDTSIILFLNKKDLFGEKIKKSPLTICFPEYTGPNTYEDAAAYIQAQFESKNRSPNKEIYCHMTCATDTNNIQVVFDAVTDIIIA
NNLRGCGLY*

$G_{\alpha oB}^{Nluc}$

MGCTLSAEERAALERSKAIEKNLKEDGISAAKDVKLLLLGAGESGKSTIVKQMKIIHEDGFSGEDVKQYKPVVYSNTIQSLAAIVRA
MDTLSGGGGSVFTLEDFVGDWRQTAGYNLDQVLEQGGVSSLFQNLGVSVTPIQRIVLSGENGLKIDIHVIIPYEGLSGDQMGQIEKIF
KVVYPVDDHHFKVILHYGTLVIDGVTPNMIDYFGRPYEGIAVFDGKKITVTGTLWNGNKIIDERLINPDGSLLFRVTINGVTGWRLCE
RILASGGGGSGIEYGDKERKADAKMVCDVVSRMEDTEPFSAELLSAMMRLWGDSGIQECFNRSREYQLNDSAKYYLDSLDRIGAA
DYQPTEQDILRTRVKTTGIVETHFTFKNLHFRLFDVGGQRSERKKWIHCFEDVTAIIFCVALSGYDQVLHEDETTNRMHESLKLFDSIC
NNKWFTDTSIILFLNKKDIFEEKIKKSPLTICFPEYTGPSAFTEAVAYIQAQYESKNKSAHKEIYTHVTCATDTNNIQFVFDAVTDVIIAK
NLRGCGLY*

$G_{\alpha z}^{Nluc}$

MGCRQSSEEKEAARRSRRIDRHLRSESQRQRREIKLLLLGTSNSGKSTIVKQMKIIHSGGFNLEACKEYKPLIIYNAIDSLTRIIRALAA
LRIDFHNPDRAYDAVQLFALTGPSGGGGSVFTLEDFVGDWRQTAGYNLDQVLEQGGVSSLFQNLGVSVTPIQRIVLSGENGLKIDIHV
IIPYEGLSGDQMGQIEKIFKVVYPVDDHHFKVILHYGTLVIDGVTPNMIDYFGRPYEGIAVFDGKKITVTGTLWNGNKIIDERLINPDG
SLLFRVTINGVTGWRLCERILASGGGGSAESKGEITPELLGVMRRLWADPGAQACFSRSSEYHLEDNAAYYLNDLERIAADYIPTVE
DILRSRDMTTGIVENKFTFKELTFKMVDVGGQRSERKKWIHCFEGVTAIIFCVELSGYDLKLYEDNQTSRMAESLRLFDSICNNNWFI
NTSLILFLNKKDLLAEKIRRIPLTICFPEYKGQNTYEEAAVYIQRQFEDLNRNKETKEIYSHFTCATDTSNIQFVFDAVTDVIIQNNLKYI
GLC*

Supplementary Fig. 12. Sequences of the constructs used for human $G\alpha_{i1}^{Nluc}$, $G\alpha_{i2}^{Nluc}$, $G\alpha_{i3}^{Nluc}$, $G\alpha_{oA}^{Nluc}$, $G\alpha_{oB}^{Nluc}$ and $G\alpha_{z}^{Nluc}$. The $G\alpha$ proteins (black), amino acid linkers (red) and Nluc (blue) are indicated.

$^{Venus}G_{\gamma 2}$

MVSKGEELFTGVVPILVELDGDVNGHKFSVSGEGEGDATYGKLTLKLICTTGKLPVPWPTLVTTLGYGLQCF
ARYPDHMKQHDFFKSAMPEGYVQERTIFFKDDGNYKTRAEVKFEGDTLVNRIELKGIDFKEDGNILGHKLE
YNYNSHNVYITADKQKNGIKANFKIRHNIEDGGVQLADHYQQNTPIGDGPVLLPDNHYLSYQSALSKDPNE
KRDHMVLLEFVTAAGITLGMDELYKKLGTMASNNTASIAQARKLVEQLKMEANIDRIKVSKAAADLMAYC
EAHAKEDPLLTPVPASENPFREKKFFCAIL*

$^{Venus}G_{\gamma 8}$

MVSKGEELFTGVVPILVELDGDVNGHKFSVSGEGEGDATYGKLTLKLICTTGKLPVPWPTLVTTLGYGLQCF
ARYPDHMKQHDFFKSAMPEGYVQERTIFFKDDGNYKTRAEVKFEGDTLVNRIELKGIDFKEDGNILGHKLE
YNYNSHNVYITADKQKNGIKANFKIRHNIEDGGVQLADHYQQNTPIGDGPVLLPDNHYLSYQSALSKDPNE
KRDHMVLLEFVTAAGITLGMDELYKKLGTMSNNMAKIAEARKTVEQLKLEVNIDRMKVSQAAAELLAFC
ETHAKDDPLVTPVPAAENPFRDKRLFCVLL*

$^{Venus}G_{\gamma 9}$

MVSKGEELFTGVVPILVELDGDVNGHKFSVSGEGEGDATYGKLTLKLICTTGKLPVPWPTLVTTLGYGLQCF
ARYPDHMKQHDFFKSAMPEGYVQERTIFFKDDGNYKTRAEVKFEGDTLVNRIELKGIDFKEDGNILGHKLE
YNYNSHNVYITADKQKNGIKANFKIRHNIEDGGVQLADHYQQNTPIGDGPVLLPDNHYLSYQSALSKDPNE
KRDHMVLLEFVTAAGITLGMDELYKKLGTMAQDLSEKDLLKMEVEQLKKEVKNTRIPISKAGKEIKEYVE
AQAGNDPFLKGIPEDKNPFKEKGGCLIS*

Supplementary Fig. 13. Sequences of the constructs used for human $^{Venus}G\gamma_2$, $^{Venus}G\gamma_8$ and $^{Venus}G\gamma_9$. The Venus (orange), amino acid linker (red) and Gγ subunit (purple) are indicated.
