# Supplementary material for: Specific pharmacological and Gi/o protein responses of some native GPCRs in neurons
Source: Nat Commun. 2024 Mar 5;15:1990. doi: 10.1038/s41467-024-46177-z (PMC10914727; doi:10.1038/s41467-024-46177-z)
Supplement: Supplementary file 3 — Reporting Summary [file 41467_2024_46177_MOESM3_ESM.pdf]

Corresponding author(s): Jean-Philippe Pin, Philippe Rondard, Jianfeng Liu

Last updated by author(s): Jan 15, 2024

Reporting Summary

Nature Portfolio wishes to improve the reproducibility of the work that we publish. This form provides structure for consistency and transparency in reporting. For further information on Nature Portfolio policies, see our Editorial Policies and the Editorial Policy Checklist.

Statistics

For all statistical analyses, confirm that the following items are present in the figure legend, table legend, main text, or Methods section.

n/a	Confirmed	
☐	☒	The exact sample size (*n*) for each experimental group/condition, given as a discrete number and unit of measurement
☐	☒	A statement on whether measurements were taken from distinct samples or whether the same sample was measured repeatedly
☐	☒	The statistical test(s) used AND whether they are one- or two-sided<br>*Only common tests should be described solely by name; describe more complex techniques in the Methods section.*
☒	☐	A description of all covariates tested
☐	☒	A description of any assumptions or corrections, such as tests of normality and adjustment for multiple comparisons
☐	☒	A full description of the statistical parameters including central tendency (e.g. means) or other basic estimates (e.g. regression coefficient) AND variation (e.g. standard deviation) or associated estimates of uncertainty (e.g. confidence intervals)
☐	☒	For null hypothesis testing, the test statistic (e.g. *F*, *t*, *r*) with confidence intervals, effect sizes, degrees of freedom and *P* value noted<br>*Give P values as exact values whenever suitable.*
☒	☐	For Bayesian analysis, information on the choice of priors and Markov chain Monte Carlo settings
☒	☐	For hierarchical and complex designs, identification of the appropriate level for tests and full reporting of outcomes
☒	☐	Estimates of effect sizes (e.g. Cohen's *d*, Pearson's *r*), indicating how they were calculated

Our web collection on statistics for biologists contains articles on many of the points above.

Software and code

Policy information about availability of computer code

Data collection	BRET measurements were acquired using Mithras LB 940 multimode microplate reader (Berthold Technologies, Bad Wildbad, Germany) with the program MikroWin, Version 4.41; or PHERAstar FS with the program PHERAstar control Version 4.00 R4.<br>Images were obtained with an Olympus FV1000 laser scanning confocal microscope.<br>Image of gel were collected using an Odyssey infrared scanner (LI-COR Biosciences, Lincoln, NE, USA) at 700nm.
Data analysis	Data were plotted and statistically analyzed using Prism (Versions 9.5.1, GraphPad Software). Dose-response experiments were analyzed using nonlinear curve fitting for the log (agonist) vs. response (three parameters) curves. Statistical analysis was performed using the Ordinary one-way ANOVA with a Dunnett's post-hoc multiple comparisons or unpaired t test (two-tailed) or paired t test. P < 0.05 was considered to be statistically significant.<br>Phylogenetic trees of human Ggamma subunits are classified in I-V groups using MegaX software. The numbers at the nodes of the branches represent the percentage of the bootstrap test (1000 replicates). Scale bar, 0.1 = 10% genetic distance.

For manuscripts utilizing custom algorithms or software that are central to the research but not yet described in published literature, software must be made available to editors and reviewers. We strongly encourage code deposition in a community repository (e.g. GitHub). See the Nature Portfolio guidelines for submitting code & software for further information.

Data

Policy information about availability of data

All manuscripts must include a data availability statement. This statement should provide the following information, where applicable:

- Accession codes, unique identifiers, or web links for publicly available datasets
- A description of any restrictions on data availability
- For clinical datasets or third party data, please ensure that the statement adheres to our policy

All the other data generated in this study are provided in the Supplementary information and source data files. The raw data and p values for all Figures and Supplementary Figures are available in Source Data files, accompanying this paper. Source data are provided with this paper. A reporting summary for this article is available as a Supplementary Information file.

Research involving human participants, their data, or biological material

Policy information about studies with human participants or human data. See also policy information about sex, gender (identity/presentation), and sexual orientation and race, ethnicity and racism.

Reporting on sex and gender	N/A
Reporting on race, ethnicity, or other socially relevant groupings	N/A
Population characteristics	N/A
Recruitment	N/A
Ethics oversight	N/A

Note that full information on the approval of the study protocol must also be provided in the manuscript.

Field-specific reporting

Please select the one below that is the best fit for your research. If you are not sure, read the appropriate sections before making your selection.

☒ Life sciences ☐ Behavioural & social sciences ☐ Ecological, evolutionary & environmental sciences

For a reference copy of the document with all sections, see nature.com/documents/nr-reporting-summary-flat.pdf

Life sciences study design

All studies must disclose on these points even when the disclosure is negative.

Sample size	Sample sizes were not predetermined by any statistical metrics, but is consistent with those of other similar reports in the literature for GPCR functional assays. Sample size for each experiments were determined based on standards for experimental cell biology, with a minimum of n = 3 biological independent replicates with sufficient reproducibility. All the presented data are mean ± SEM or representative results for at least three experiments performed independently in triplicate or quadruplicate. Information on the number of replicates and independent experiments that were performed for each measurement is provided in the manuscript.
Data exclusions	No data were systematically excluded. All raw data were provided in the source data. Some individual outliers in the triplicate or quadruplicate which was obviously vary from the others, were excluded from the analysis (in most cases, less than three points per 96-well plates were excluded).
Replication	Number of independent experiments and replicates are indicated in the legends to the figures. Experimental findings were reliably reproduced.
Randomization	No randomization was attempted or needed. Randomization was not necessary as the independent variables to be tested were sufficient for the functional interpretation within this study.
Blinding	Blinding was not applicable to this study. All experimental data were acquired using automated equipment and analyzed using computational softwares, eliminating human error and bias.

Reporting for specific materials, systems and methods

We require information from authors about some types of materials, experimental systems and methods used in many studies. Here, indicate whether each material, system or method listed is relevant to your study. If you are not sure if a list item applies to your research, read the appropriate section before selecting a response.

Materials & experimental systems

n/a	Involved in the study
☐	☒ Antibodies
☐	☒ Eukaryotic cell lines
☒	☐ Palaeontology and archaeology
☐	☒ Animals and other organisms
☒	☐ Clinical data
☒	☐ Dual use research of concern
☒	☐ Plants

Methods

n/a	Involved in the study
☒	☐ ChIP-seq
☒	☐ Flow cytometry
☒	☐ MRI-based neuroimaging

Antibodies

Antibodies used	Anti-GFP mouse mAb (ab1218, 1:200, Abcam, Shanghai, China)<br>Alexa Fluor® 488 AffiniPure Donkey Anti-Mouse IgG (H+L) (715-545-150, 1:500, Jackson ImmunoResearch, Shanghai, China)<br>Anti-GABAB1 mouse mAb (ab55051, 1:1000, Abcam, Shanghai China)<br>Anti-CNR1 Rabbit pAb (A1447, 1:1000, ABclonal Technology., Wuhan, China)<br>Anti-ADRA2A rabbit pAb (A2809, 1:1000, ABclonal Technology., Wuhan, China)<br>Anti-GNB1 Rabbit pAb (A1867, 1:1000, ABclonal Technology., Wuhan, China)<br>Anti-GNB2 Rabbit mAb (A9643, 1:1000, ABclonal Technology., Wuhan, China)<br>Anti-GNB3 Rabbit pAb (A1387, 1:1000, ABclonal Technology., Wuhan, China)<br>Anti-GNB5 Rabbit pAb (A4447, 1:1000, ABclonal Technology., Wuhan, China)<br>Anti-beta-actin antibody (KM9001T, 1:3000, Sungene Biotech., Tianjin, China)<br>Anti-mouse IgG DyLight 800 4X PEG conjugated secondary antibodies (#5257, 1:20000, Cell Signaling Technology, Shanghai, China)<br>Anti-rabbit IgG DyLight 800 4X PEG conjugated secondary antibodies (#5151, 1:20000, Cell Signaling Technology, Shanghai, China)
Validation	Commercial antibodies used in this study are widely used and have been validated by the respective manufacturer.<br>GFP antibody Abcam Cat#ab1218: 23 citations reported on manufacturer's website (https://www.abcam.cn/products/primary-antibodies/gfp-antibody-9f9f9-ab1218.html)<br>GABAB1 antibody Abcam Cat#ab55051: 2 citations reported on manufacturer's website (https://www.abcam.cn/products/primary-antibodies/gaba-b-receptor-1-antibody-2d7-ab55051.html)<br>Anti-ADRA2A ABclonal Cat#A2809: 1 citations reported on manufacturer's website (https://abclonal.com.cn/catalog/A2809)<br>Anti-GNB1 ABclonal Cat#A1867: Validated by company (https://abclonal.com.cn/catalog/A1867)<br>Anti-GNB2 ABclonal Cat#A9643: Validated by company (https://abclonal.com.cn/catalog/A9643)<br>Anti-GNB3 ABclonal Cat#A1387: 1 citations reported on manufacturer's website (https://abclonal.com.cn/catalog/A1387)<br>Anti-GNB5 ABclonal Cat#A4447: 1 citations reported on manufacturer's website (https://abclonal.com.cn/catalog/A4447)<br>Anti-beta-actin antibody Sungene Biotech Cat#KM9001T: Validated by company (http://www.sungenebiotech.com/index.php?m=Product&a=product_xq&catid=2&proid=53&prid=289&pid=717&id=1548)<br>Anti-mouse IgG DyLight 800 4X PEG conjugated secondary antibodies Cell Signaling Technology Cat#5257: 129 citations reported on manufacturer's website (https://www.cellsignal.cn/products/secondary-antibodies/anti-mouse-igg-h-l-dylight-8482-800-4x-peg-conjugate/5257)<br>Anti-rabbit IgG DyLight 800 4X PEG conjugated secondary antibodies Cell Signaling Technology Cat#5151: 244 citations reported on manufacturer's website (https://www.cellsignal.cn/products/secondary-antibodies/anti-rabbit-igg-h-l-dylight-800-4x-peg-conjugate/5151)<br>Alexa Fluor® 488 AffiniPure Donkey Anti-Mouse IgG (H+L) Jackson ImmunoResearch Cat#715-545-150: 666 citations reported on manufacturer's website (https://www.jacksonimmuno.com/catalog/products/715-545-150)

Eukaryotic cell lines

Policy information about cell lines and Sex and Gender in Research

Cell line source(s)	HEK293 cells (ATCC, CRL-1573, lot: 3449904) were used.
Authentication	HEK293 cell line was obtained from ATTC and used without further authentication.
Mycoplasma contamination	HEK293 cells were tested monthly in laboratory and no mycoplasma contamination was found, which was described in the manuscript.
Commonly misidentified lines (See ICLAC register)	No commonly misidentified cell lines were used in this study.

Animals and other research organisms

Policy information about studies involving animals; ARRIVE guidelines recommended for reporting animal research, and Sex and Gender in Research

Laboratory animals	One-week-old newborn Kunming mice were used for primary cerebellar granule neuronal cultures and embryonic day 17.5 Kunming mice were used for cortical and hippocampal neuronal cultures. The mice were raised in a specific pathogen free (SPF) environment with an ambient temperature of 18-22°C, a humidity of 50%-60%, and a 12 h light-dark cycle.
Wild animals	The study did not involve wild animals.
Reporting on sex	Both males and females were used (no need for gender analysis).
Field-collected samples	The study did not involve samples collected from field.
Ethics oversight	This project followed the Animal Welfare Body and was approved by the internal Ethic Committee of the College of Life Science and Technology, Huazhong University of Science and Technology, Wuhan, China.

Note that full information on the approval of the study protocol must also be provided in the manuscript.

Plants

Seed stocks	N/A
Novel plant genotypes	N/A
Authentication	N/A
